# High daily energy expenditure of Tuvan nomadic pastoralists living in an extreme cold environment

**DOI:** 10.1038/s41598-022-23975-3

**Published:** 2022-11-22

**Authors:** Adam J. Sellers, Dolaana Khovalyg, Guy Plasqui, Wouter van Marken Lichtenbelt

**Affiliations:** 1 Department of Nutrition and Movement Sciences, School of Nutrition and Translational Research in Metabolism (NUTRIM), Maastricht University, Maastricht, The Netherlands; 2 Laboratory of Integrated Comfort Engineering (ICE), École Polytechnique Fédérale de Lausanne (EPFL), Lausanne, Switzerland

**Keywords:** Physiology, Metabolism

## Abstract

Research investigating thermoregulatory energy costs in free-living humans is limited. We determined the total energy expenditure (TEE) of Tuvan pastoralists living in an extreme cold environment and explored the contribution of physical activity and cold-induced thermogenesis. Twelve semi-nomadic pastoralists (47 ± 8 years, 64 ± 8 kg) living under traditional circumstances, in Tuva, south-central Siberia, Russia, were observed during two consecutive 6-day periods in winter. TEE was measured via the doubly labelled water technique. Skin and ambient temperatures, and physical activity were continuously monitored. The outdoor temperature during the observation period was − 27.4 ± 5.4 °C. During the daytime, the participants were exposed to ambient temperatures below 0 °C for 297 ± 131 min/day. The Tuvan pastoralists were more physically active compared to western populations (609 ± 90 min/day of light, moderate, and vigorous physical activity). In addition, TEE was 13.49 ± 1.33 MJ/day (3224 ± 318 kcal/day), which was significantly larger by 17% and 31% than predicted by body mass, and fat-free mass, respectively. Our research suggests the daily cold exposure combined with high levels of physical activity contributed to the elevated TEE. Future research should reconsider the assumption that energy costs due to thermoregulation are negligible in free-living humans.

## Introduction

This study investigates the energetics of traditionally living, semi-nomadic, Tuvan livestock herders during winter. There are few studies performed with traditional living populations that live in, and are actually exposed to low outdoor temperatures. Moreover, this traditional lifestyle is gradually disappearing. Interest in this study is fuelled by the relatively recent insights that, in addition to physical activity, exposure to cold may significantly affect energy metabolism and metabolic health^1,2^.

Energy expenditure and energy intake are essential components of energy balance. Established main determinants of total energy expenditure (TEE) include body mass, body composition, food intake, and physical activity^3^. The effect of thermoregulation on TEE has received much less attention, even though cold-induced thermogenesis is evident in humans^4^. Cold exposure can increase an individual’s energy expenditure via shivering and non-shivering mechanisms^5–7^. Indeed, maximal shivering acutely increased resting oxygen consumption by 4.9 times^8^, whereas a 3-h mild cold exposure increased energy expenditure by 7% in the summer and by 11.5% in the winter^9^. Even 24-h energy expenditure was increased by 6%^10^ and 7%^11^ during prolonged mild cold conditions. This research demonstrates the potential of cold to increase daily energy expenditure.

As physical activity is a part of daily life, the interaction between physical activity and cold-induced thermogenesis should be considered. Laboratory studies show that exercising in colder water increases oxygen uptake compared to exercising in warmer water^12,13^. Exercising at a higher intensity during cold exposure can attenuate, albeit not always completely, cold-induced thermogenesis^13^. Thus, walking in a cold, rather than in a more thermoneutral, environment increases oxygen consumption by 25% when walking at a low intensity, and by 6% when walking at a higher intensity^14^. Research performed outside the laboratory, under free-living conditions, also suggests an important interaction between cold exposure and physical activity^15^. As cold exposure has the potential to increase metabolic rate, even when being physically active, the general assumption that thermoregulatory energy costs are negligible in free-living humans should be questioned.

Readily available energy-dense foods, labour-saving devices, affordable transport, and increased sedentary time are contributors to obesity^16^. It was assumed that populations from developed countries had lower physical activity energy expenditures than individuals from developing countries, hence explaining the greater obesity prevalence in developed countries^16^. Conversely, neither TEE adjusted for weight and age, nor physical activity level (PAL = TEE/REE (resting energy expenditure)), were reported to be significantly different between populations from developing and developed countries, thus suggesting a minimal role of physical activity energy expenditure in obesity at a population level^17^. However, in addition to a country’s development status, factors such as lifestyle and environmental conditions such as temperature should be considered. This is because, in addition to activity-induced energy expenditure, cold-induced thermogenesis is also variable and can lead to significant energy costs. Therefore, when studying free-living energetics, especially in cold environments, it is relevant to consider both energy expenditure due to physical activity and thermoregulation.

The lifestyle of an individual affects daily energy costs, with more physically active individuals generally having greater energy demands than sedentary individuals^3^. Professional endurance cyclists during a 3-week multi-stage race had a TEE of 32.3 ± 3.4 MJ/day and a PAL of 4.37 ± 0.43^18^. These levels are among the highest reported in the literature and may not be sustained for extended periods because of a negative energy balance. Data derived from a large data set showed that the TEE and PAL of similar aged males who engaged in more normal daily activities were 13.2 ± 3.8 MJ/day and 1.80 ± 0.39, respectively^19^. Traditional, more natural living populations such as the Hadza, that follow a hunter-gatherer lifestyle, have a similar TEE corrected for body mass when compared to populations from market economies^20^ and developed countries^21^. However, populations with a farming lifestyle do have larger TEE compared to populations from market economies^20^, thus highlighting nuances between populations with the effect of lifestyle on energy demands. The majority of research investigating daily energy requirements has taken place in warm and temperate environments, whereas research on free-living individuals in a cold environment is limited.

Many studies on individuals living in cold environments focussed on REE^22,23^, and thermoregulatory responses in the Saami^24,25^, the Yakut of Siberia^26^, and reindeer herders of Finland^27^, whereas studies on TEE are scarce. Surprisingly, the Yakut males and females of Siberia had a TEE comparable to western populations^28^. The Yakut traditionally relied on subsistence activities such as pastoralism, hunting, and fishing, whereas nowadays they rely on a mixture of subsistence and more modernised activities^28^. The participation in subsistence activities and TEE were positively correlated^28^, thus suggesting that the more traditional lifestyle increased TEE, likely by costs of physical activity or potentially, thermoregulation. Reindeer herders, during a very active period, had a greater TEE than populations of market economies and those with a hunter-gatherer lifestyle^29^. The reindeer herders’ high TEE was mainly attributed to their physical activity^29^, although the mean temperature during the month of observation was 1 °C, thus, some cold-induced thermogenesis may have contributed to the energy demands. In line, military personnel do have greater energy expenditures when active in a cold environment^30^ compared to warmer and more sedentary conditions^31^. However, the actual exposure of the participants to the cold and the contribution of cold-induced thermogenesis to TEE in the studies discussed above are unknown.

In the current study, we investigated the TEE, thermoregulation, and physical activity of free-living nomadic pastoralists in Tuva (south-central Siberia, Russia), during winter. The pastoralists lived in yurts and practised animal husbandry, which required the regular herding of livestock for many hours per day in an extreme cold environment. Total energy expenditure, resting energy expenditure, physical activity, skin temperature, and the ambient temperature of the individuals were measured. The aims of this paper were to measure the energetics of these people and determine the contribution of physical activity and cold-induced thermogenesis to TEE. Additionally, the results are compared to western populations.

## Results

### General lifestyle of the participants

After the Perestroika in Tuva, many former nomadic pastoralists returned to traditional husbandry practices, which included living in yurts and seasonal migration. The Tuvan yurts are round (mean diameter 5.6 m). In the centre is a cast-iron stove for heating and cooking. Around the inside perimeter of the yurt are a bed, extra bed, furniture for storage, and sometimes a temporary stall for livestock. Seasonal migration varied from 2 to 6 times per year in the current sample, although no migration took place during the observation period. With respect to the ownership of the 8 yurts under study, four owned a car and/or a motorcycle. Two yurts had television; no cameras, computers, stereos, or microwave ovens were owned. There was a large difference between the number of livestock per family. Some households combined their livestock and took shifts in herding. Thus, some families had only 83 animals, while others owned over 1250. The number of cows per yurt ranged from 13 to 200, horses from 0 to 100, sheep from 30 to 800, and goats from 40 to 300.

The participants (Table 1) reported that they usually woke up between 05:00 and 07:00. Morning activities consisted of firing up the stove, breakfast, and tending to livestock. During the afternoon, individuals could be within the vicinity of their yurt and taking care of the livestock, cleaning the yurt, or maintaining the shelters of livestock. Alternatively, individuals could be herding livestock for 5–10 h per day. Generally, the males performed the majority of the herding whereas the females maintained the yurt and stables. The longer herding duration was usually completed in the summer than the winter. Some individuals rode horses during the herding. By the end of the afternoon, individuals returned livestock to their shelters, had dinner, and rested in their yurt. Eating frequency was usually 2–3 times per day. The diet consisted mainly of cow, sheep, and horse meat, noodles, dumplings, noodle soup, bread, fried flat bread, butter, and salted milk tea. Individuals went to bed between 21:00 and 24:00. In addition, participants reported they fished, and gathered produce such as mushrooms and wild berries from taiga in the summer, although these activities were only performed on a few occasions for each individual.

**Table 1:** Participant characteristics.

Characteristic	Total (n = 12)	Males (n = 9)	Females (n = 3)
Age (years)	47 ± 8	45 ± 8	51 ± 7
Height (m)	1.65 ± 0.1	1.69 ± 0.07	1.52 ± 0.04
Body mass (kg)	64.2 ± 7.7	64.7 ± 7.9	62.8 ± 8.5
BMI (kg/m^2^)	23.8 ± 3.2	22.7 ± 1.9	27.4 ± 4.1
Body fat (%)	26.6 ± 8.9	22.5 ± 5.7	38.9 ± 1.5
Fat mass (kg)	17.2 ± 6.4	14.8 ± 5.2	24.5 ± 4
Fat-free mass (kg)	47 ± 6.7	49.8 ± 4.4	38.3 ± 4.7
Total body water (L)	34.3 ± 4.9	36.4 ± 3.2	28 ± 3.4

Values are mean ± SD.

**Figure 1:**
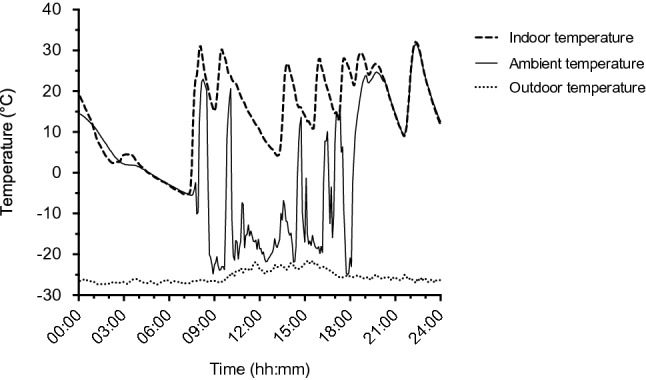
Example 24-h profiles of environmental temperatures. Indoor temperature was measured inside a yurt. Outdoor temperature was measured outside the same yurt. A participant’s (male, 58 years) ambient temperature was measured with a temperature sensor attached to the outer surface of their clothing. Sampling rate was 5 min.

A representative example of one male participant shows the thermal conditions that the participant was exposed to (ambient temperature) were highly variable, depending on whether the person was inside or outside the yurt (Fig. 1).

### Environmental temperatures during the observation periods

The mean outdoor air temperature, over 12 days was − 27.4 ± 5.4 °C (range: − 38.4 °C to − 15.4 °C). The mean outdoor temperature during the day (07:00 to 19:00) of period 1 was significantly lower than period 2 (− 28.2 ± 4.3 °C vs. − 22.3 ± 4.6 °C, P < 0.001). The mean outdoor temperature during the night (19:00 to 07:00) was also significantly lower during period 1 than period 2 (− 32.9 ± 2.3 °C vs. − 26.0 ± 3.3 °C, P < 0.001). The mean temperature inside one yurt, next to the bed, over a period of 7 days was 15.5 ± 10.9 °C (range: − 7.8 °C to 43.3 °C). There were no significant differences between period 1 and period 2 with respect to the participants’ mean ambient temperature (1.9 ± 6.9 °C vs. 3.9 ± 5.9 °C, P = 0.216), and the duration exposed to below 0 °C during the day (1786 ± 774 min vs. 1782 ± 906 min, P = 0.980).

### Skin temperatures

**Figure 2:**
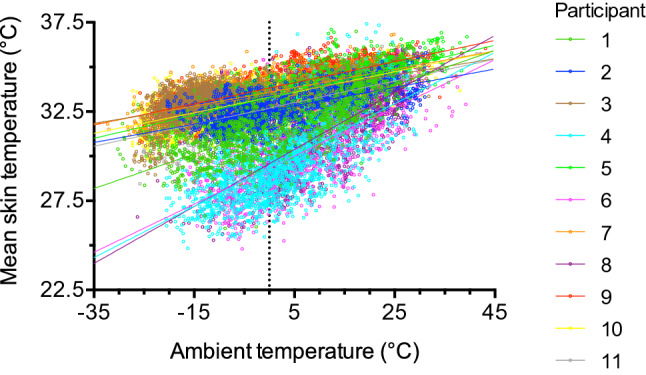
The relationship between the participant’s ambient temperature and mean skin temperature during the day (07:00–19:00) of the whole period. One subject’s data were excluded due to non-wear of the clothing temperature sensor, therefore n = 11 (8 males and 3 females). Participant numbers 4, 6, and 8 are females.

**Figure 3:**
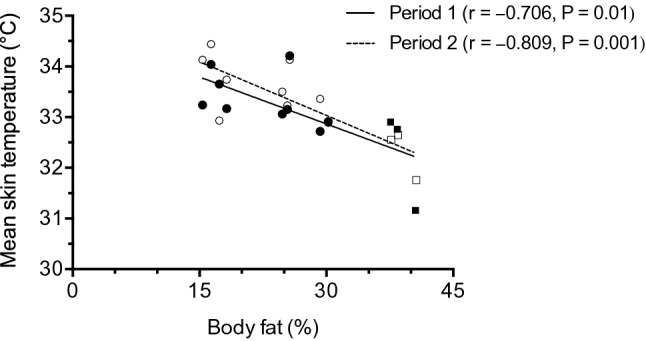
The relationship between the participant’s body fat % and their mean 24-h skin temperature during period 1 (filled markers) and period 2 (unfilled markers). Circles denote males and squares denote females. The r value is the result of the Pearson correlation.

The participants’ mean skin temperature (Table 2) was similar during period 1 and period 2 (33.1 ± 0.8 °C vs. 33.3 ± 0.8 °C, P = 0.172). There was a significant relationship between the participant’s daytime mean skin temperature and their ambient temperature (Fig. 2). Including subject as a random effect intercept significantly improved the model (P < 0.001). At an ambient temperature of 0 °C, males had a predicted mean skin temperature (mean ± SE) of 33.1 ± 0.2 °C whereas the predicted mean skin temperature of females was 3.6 ± 0.4 °C less (P < 0.001) than males. The mean skin temperature of males changed by 0.06 ± 0.001 °C (P < 0.001) for each 1 °C change in ambient temperature, whereas the mean skin temperature of females changed by 0.088 ± 0.002 °C (P < 0.001) more than males. Mean 24-h skin temperature during both periods 1 and 2 were each negatively correlated with body fat % (Fig. 3).

**Table 2:** Skin temperatures during the day and night of both periods.

Variable	Period 1	Period 2
Mean skinT day (°C)	32.4 ± 1.3***	32.6 ± 1.3**
Mean skinT night (°C)	33.8 ± 0.5***	33.9 ± 0.5**
ChestT day (°C)	33.0 ± 1.8**	33.2 ± 1.6***
ChestT night (°C)	34.6 ± 0.6**^#^	34.8 ± 0.6***^#^
Upper armT day (°C)	33.1 ± 0.9	33.1 ± 1.6
Upper armT night (°C)	33.4 ± 0.7	33.5 ± 0.8
ThighT day (°C)	30.0 ± 2.2***^#^	30.6 ± 2.2***^#^
ThighT night (°C)	33.5 ± 0.7***	33.7 ± 0.5***
Lower legT day (°C)	32.8 ± 0.9*	33.1 ± 1.1
Lower legT night (°C)	33.4 ± 0.6*	33.5 ± 0.3

Values are mean ± SD. “T” denotes temperature. “Day” includes data collected between 07:00 and 19:00 and “night” includes data collected between 19:00 and 07:00. Period 1 and period 2 are 6 days each. For all skin temperature parameters, n = 12. Data were analysed with paired t-tests. Significant differences between periods 1 and 2 are indicated by: ^#^P < 0.05. Significant differences during the day and the night, within periods, are indicated by: *P < 0.05, **P < 0.01 and ***P < 0.001.

### Physical activity

During the whole observation period, the participants had a mean vector magnitude of 989 ± 228 CPM (counts per min) /day (range: 683–1299 CPM/day). The mean durations performing different physical activity intensities were: sedentary (including sleeping), 831 ± 90 min/day (range: 673–980 min/day); light activity, 475 ± 91 min/day (range: 370–632 min/day); moderate activity, 125 ± 60 min/day (range: 33–224 min/day); and vigorous activity, 8 ± 11 min/day (range: 0–32 min/day).

Physical activity tended to be lower during period 1 than period 2 (944 ± 192 vs. 1035 ± 287 CPM/day, P = 0.099), which may be due to the fact that individuals performed 31 min less per day of moderate physical activity during period 1 than period 2 (110 ± 51 min/day vs. 141 ± 77 min/day, P = 0.056). The durations spent being sedentary (including sleeping) (835 ± 92 min/day vs. 828 ± 99 min/day, P = 0.715), performing light physical activity (489 ± 94 min/day vs. 462 ± 98 min/day, P = 0.164), and performing vigorous physical activity (7 ± 10 min/day vs. 9 ± 12 min/day, P = 0.183) were similar during periods 1 and 2.

### Resting energy expenditure, total energy expenditure, and physical activity level

Resting energy expenditure was 7.31 ± 1.17 MJ/day which is significantly higher than predicted by age, body mass, and height according to the Harris and Benedict^32^ (6.05 ± 0.65 MJ/day, P = 0.005) and Mifflin^33^ equations (5.87 ± 0.76 MJ/day, P = 0.003). Resting energy expenditure was also higher than predicted by fat-free mass^34^ (5.63 ± 0.62 MJ/day, P < 0.001). Resting energy expenditure was not related to body mass or body composition.

Total energy expenditure during the whole observation period was 13.49 ± 1.33 MJ/day (range: 11.11 MJ/day to 15.15 MJ/day) and tended to be larger during period 1 than period 2 (13.99 ± 1.86 vs. 12.98 ± 1.33 MJ/day, P = 0.086). Total energy expenditures of males and females were 13.58 ± 1.62 MJ/day and 13.21 ± 1.94 MJ/day, respectively. Total energy expenditure was not related to body mass, physical activity, skin temperature, day ambient temperature, or the duration exposed to below 0 °C. Additionally, the difference in TEE between periods 1 and 2 was not related to differences in physical activity, skin temperature, day ambient temperature, or the duration exposed to below 0 °C.

**Figure 4:**
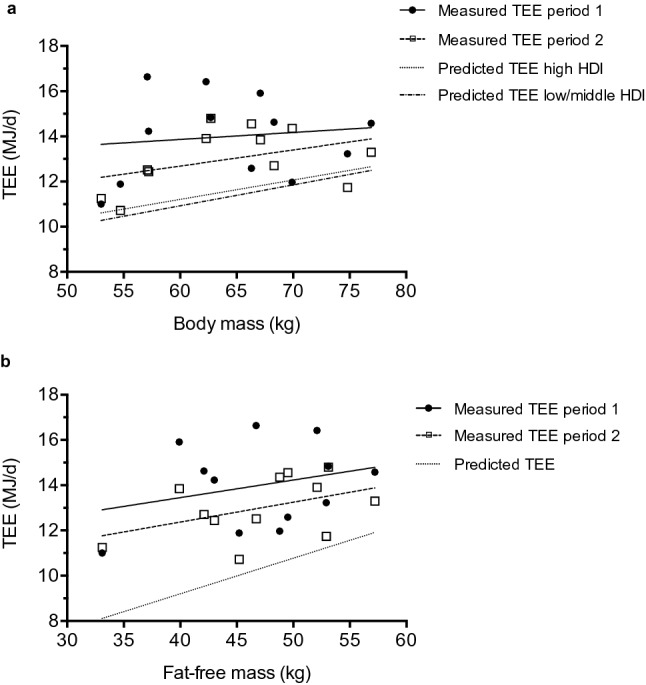
Measured total energy expenditure (TEE) and TEE predicted by body mass (**a**) and fat-free mass (**b**). Predicted TEE high or low/middle HDI refers to the prediction equation created with countries rated as either high, or low/middle on the human development index (HDI).

One-way repeated measures ANOVAs indicate a significant difference between measured TEE and TEE predicted by body mass (P < 0.001) and fat-free mass (P < 0.001). Post-hoc tests identified that measured TEE during the whole period (13.49 ± 1.33 MJ/day), period 1 (13.99 ± 1.86 MJ/day), and period 2 (12.98 ± 1.33 MJ/day) were greater than TEE predicted by body mass (11.57 ± 1.16 MJ/day) based on developed countries (Fig. 4a, all P < 0.006), TEE predicted by body mass (11.32 ± 1.24 MJ/day) based on developing countries (Fig. 4a, all P < 0.003) and TEE (10.30 ± 1.06 MJ/day) predicted by fat-free mass (Fig. 4b, all P < 0.001).

The Tuvan pastoralists’ PAL during the whole period was 1.89 ± 0.34 (range: 1.57–2.74). Physical activity levels were similar during period 1 and period 2 (1.95 ± 0.31 vs. 1.83 ± 0.42, P = 0.108). There were no relations between PAL and physical activity, skin temperature, day ambient temperature, or the duration exposed to below 0 °C.

## Discussion

The current research investigated the TEE, physical activity, and thermoregulation of nomadic pastoralists, living traditionally, in Tuva during two consecutive 6-day periods in winter when the mean outdoor temperature was − 27.4 ± 5.4 °C. This study uniquely combines measurements of TEE, physical activity, skin temperature, and the ambient temperature of people living in an extreme cold environment. The Tuvan pastoralists were frequently exposed to the extreme cold and had high physical activity levels. Total energy expenditure of the Tuvan pastoralists was significantly greater than predicted by body mass, and fat-free mass. It is likely that the combination of daily cold exposure and high levels of physical activity contributed to the elevated TEE.

The average body mass of the Tuvan pastoralists was relatively low. Despite this, TEE of both males (13.58 ± 1.62 MJ/day) and females (13.21 ± 1.94 MJ/day) were larger than the average TEE of similar aged adults^19^ (males: 12.68 ± 2.39 MJ/day; females: 9.75 ± 1.59 MJ/day). When compared to the Yakut, also of Siberia, the Tuvan pastoralists had a higher TEE^28^. This can be attributed to the Yakut living under less traditional circumstances and in more modern housing conditions. The Tuvans had a lower TEE compared to reindeer herders from subarctic Finland^29^. The reindeer herders study, however, was conducted during the annual herd round-up, which involves a large amount of physical activity and is therefore unlikely to be representative of general energy demands. Additionally, the reindeer herders’ body mass was 22.5 kg greater than the current participants. Taking body mass into account, the Tuvans’ TEE was 1.92 MJ/day (17%) and 2.17 MJ/day (19%) larger than predicted by body mass based on equations from developed and developing countries^17^, respectively. Furthermore, measured TEE was 3.19 MJ/day (31%) greater than TEE predicted by fat-free mass^19^. The elevated TEE in the current study is in contrast to the reported no difference in TEE of populations from developed countries and developing countries^17^. Thus, our results demonstrate that not all populations have a similar TEE. Below, we explore potential explanations for the elevated TEE in the Tuvan pastoralists.

The REE of the nomadic pastoralists determined with indirect calorimetry was greater than predicted by age, body mass, and height, and also larger than predicted by fat-free mass. This finding is similar to the elevated basal energy expenditure reported in other individuals living in cold environments, such as the Yakut from Siberia^35^ and other circumpolar populations^22^, and may contribute to the increased TEE. However, it should be noted that part of the elevated REE observed in the current study may be attributed to the use of a face-mask system^36^, and the duration of the fast was only 4 h, thus some diet-induced thermogenesis may have remained^37,38^. Despite the REE likely being overestimated, thus resulting in an underestimated PAL, the PAL of the Tuvan males (1.91 ± 0.39) was greater than previously reported for a similar age group (1.77 ± 0.28)^19^. The Tuvan females (1.81 ± 0.11) also had a higher PAL than previously reported for a similar age group (1.71 ± 0.16). This elevated PAL suggests the Tuvan pastoralists have greater energy costs for processes other than REE compared to other similar aged adults, such as activity and/or cold-induced thermogenesis. Additionally, the Tuvan pastoralists had a greater PAL compared to the Yakut^28^. One reason for the higher PAL may be the lower mean outdoor temperature in Tuva compared to the Yakut (− 27.4 °C vs. 12.6 °C), which could increase thermoregulatory energy costs. Also, we specifically recruited individuals who practised pastoralism, which requires physical labour.

Independent from the determination of PAL, we assessed physical activity more directly by accelerometry. These results confirm high levels of daily physical activity. The Tuvans performed 609 ± 90 min/day of light, moderate, and vigorous activity during the observation period, of which they performed 133 ± 66 min/day of moderate to vigorous physical activity. These values are higher than the Yakut males and females who had moderate to vigorous physical activity durations of 60 min/day and 36 min/day, respectively^39^. They are also greater than reported for adults in the United States^40^ and the Netherlands^41^, of which both studies included an Actigraph accelerometer and used the same physical activity intensity cut-off values as the current study. The Tuvan pastoralists are more physically active than western populations and the Siberian Yakut, which is likely a result of their traditional pastoralist lifestyle and may explain their greater than predicted TEE.

During the whole period, TEE or PAL were not significantly related to mean vector magnitude as determined by accelerometry. This is in contrast to many, although not all, of the studies using both accelerometry and the doubly labelled water method^42,43^. Several factors can impact the relationships between physical activity and TEE or PAL. In the present study, some individuals rode horses. Thus, movement of the horse may have been registered by accelerometry. Additionally, differences in the amount of upper-body activity performed, load carried, or ground surface, as also suggested by others during free-living conditions^44^, may have disturbed the physical activity and energy expenditure relationship. Finally, cold-induced thermogenesis may have altered the relationship between physical activity and TEE or PAL.

The Tuvan pastoralists lived and performed their daily activities in a cold environment, as the mean outdoor temperature was − 27.4 ± 5.4 °C. During the daytime, participants were exposed to temperatures below 0 °C for 297 ± 131 min/day. Daytime skin temperatures were lower than at night. During the night, mean skin temperatures of the chest, upper arm, thigh, and lower leg were within 1 °C of skin temperatures previously recorded in resting males during comfortable laboratory conditions^45^. This suggests the Tuvan pastoralists were generally thermoneutral at night, and they were colder during the day. Additionally, when the participants were exposed to low ambient temperatures, they were also colder. This can be derived from the positive relationship between mean skin temperature and the participant’s ambient temperature (Fig. 2). Females had a lower mean skin temperature than males at lower ambient temperatures. This result is in agreement with others who also reported lower skin temperatures in females than males^46^. The effect of sex on mean skin temperature could be explained by differences in body fat percentage as suggested by the negative relationships in the current study (Fig. 3). In agreement, others also suggest that adiposity can alter regional body temperatures^47,48^. The Tuvan pastoralists were exposed to very low environmental temperatures, which reduced their skin temperature; thus, it is probable that energy costs associated with cold-induced thermogenesis also contributed to the greater than predicted TEE.

To study whether cold affected TEE during the whole period, the relationships between indicators of cold exposure with TEE and PAL were examined. Mean skin temperature was not significantly related to TEE or PAL. However, certain factors should be considered when using mean skin temperature. Firstly, skin temperatures are affected by an individual’s body composition^46^. Secondly, physical activity impacts skin temperature^14^. Thirdly, clothing insulation affects the skin temperature during both resting and walking at − 15 °C^49^. Therefore, these factors may disturb the relationship between skin temperature and TEE or PAL. Moreover, mean skin temperature was determined with a weighted formula that included measures of the chest, upper arm, thigh, and lower leg temperatures^50^. This four-point formula has been recommended for field measurements^51^. However, formulas including less than seven points are suggested to be inaccurate in cool environments^52^. Thus, a relation may have been observed if more skin measurement sites were included.

Mean day ambient temperature and the duration exposed to below 0 °C during the day were used as additional indicators of cold exposure. No significant relationships were found between these indicators and TEE or PAL. This could be due to the individualised physiological responses to cold exposure. During cold exposure, some individuals have a greater insulative response whereas others have a more pronounced metabolic response which is characterised by greater cold-induced thermogenesis^10^. Body fat is one factor responsible for attenuating cold-induced thermogenesis^53,54^. Thus, when considering the large range in body fat percentage in the current study (15–41%), it is possible that the individualised responses to cold exposure weakened the relationships between the indicators of cold exposure and TEE or PAL. Moreover, cold-induced thermogenesis may have been attenuated by increases in physical activity^13,14^ thus distorting the relationship between cold exposure and TEE.

Finally, the combination of physical activity and indicators of cold exposure in relation to TEE or PAL were investigated. The combination of mean vector magnitude with either mean skin temperature, mean day ambient temperature, or mean duration exposed to below 0 °C, were not significantly related to TEE or PAL. Next, we explored further the differences, within subjects, between the two periods.

Mean outdoor temperatures during the day and night of period 1 were lower than during period 2. Mean skin temperature was similar during period 1 and period 2, although, day thigh temperature and night chest temperature were lower during period 1. In line with this, TEE tended to be greater during period 1 than period 2. Interestingly, accelerometry revealed higher levels of physical activity during period 2. The lower temperatures observed during period 1 indicate that participants were cooler. This, combined with the lower amount of physical activity during period 1 may suggest that the increased TEE during period 1 was indeed due to increased thermoregulatory energy demands.

Further regression analyses revealed that the combination of vector magnitude period change and mean day ambient temperature period change explained 43% (P = 0.108) of the variation in the difference between periods in TEE. The 43% variation explained within individuals is greater than the 8% (P = 0.716) explained by the combination of mean vector magnitude and mean day ambient temperature during the whole period. This suggests that the combination of physical activity and cold exposure affects energy expenditure. However, due to the small sample size, statistical power was low. Nonetheless, others also attributed an elevated TEE in cold environments, compared to temperate and hot environments, to increased energy costs associated with physical activity and thermoregulation^15^.

Generally, the contribution of thermoregulatory energy costs to TEE in humans has received minimal attention and is sometimes assumed to be negligible. This could have important implications for research investigating the effect of energy expenditure on obesity^17,55^, as there may be differences in physiological responses, such as appetite and energy intake, when the same amount of energy is expended via cold-induced thermogenesis or physical activity. Recent research suggests that environmental temperature does not affect the TEE of adults living in the United States^56^. However, the actual cold exposure experienced by the participants was unknown, the lower outdoor temperatures were limited to − 10 °C, and people were likely exposed predominantly to indoor temperatures. In the present study, we also did not find a significant relationship between TEE and indicators of the pastoralist’s actual cold exposure, although in this case, this is likely due to differences in clothing, body composition, and physical activity.

The present results demonstrate the highly active, cold exposed, Tuvan pastoralists have a greater than predicted TEE. Notably, the greater than predicted TEE of the Tuvan pastoralists is in contrast to the reported similarities between the TEE of populations from developed and less developed countries^17^, including another physically active, traditional population (Hazda, hunter-gatherers)^20,21^. This difference may be attributed to the higher energy costs associated with physical activity and cold-induced thermogenesis in the Tuvan pastoralists. The elevated TEE in the present study also contrasts with the Constrained Total Energy Expenditure model^57^ which suggests that above moderate physical activity levels, TEE plateaus to maintain TEE within a narrow range. Considering the interaction between physical activity and thermoregulation^13,14^, it should be considered the higher physical activity may have simply reduced thermoregulatory energy costs^57^. It is thus recommended to consider cold-induced thermogenesis in future research that aims to develop and refine models on energy expenditure and obesity.

A limitation of the research is the low number of females compared to males. Additionally, due to the field work schedule, it was not possible to perform the REE measurement under more strict conditions to determine basal energy expenditure. Therefore, caution is advised when interpreting the measured REE as it is likely to be overestimated compared to a true basal energy expenditure measurement. A consequence of this is that the PAL in the current study may have been underestimated, and the pastoralists actually expended relatively more energy during the observation period on processes other than their REE.

In conclusion, the Tuvan nomadic pastoralists had an active lifestyle and were exposed to an extreme cold environment. The Tuvans were physically active for 10 h/day, which involved activities such as herding livestock. During the daytime, they were exposed to temperatures below 0 °C for 5 h/day. Total energy expenditure, measured by doubly labelled water, was 17% and 31% greater than predicted by body mass, and fat-free mass, respectively. The large amount of physical activity, assessed by accelerometry, and cold exposure, determined via skin and environmental temperatures, are both likely to contribute to the greater than predicted TEE. Future research investigating daily energy expenditure in humans should reconsider the assumption that energy costs due to thermoregulation are negligible, especially for populations that live, and are active, in a cold environment.

## Methods

### Study approval

**Figure 5:**
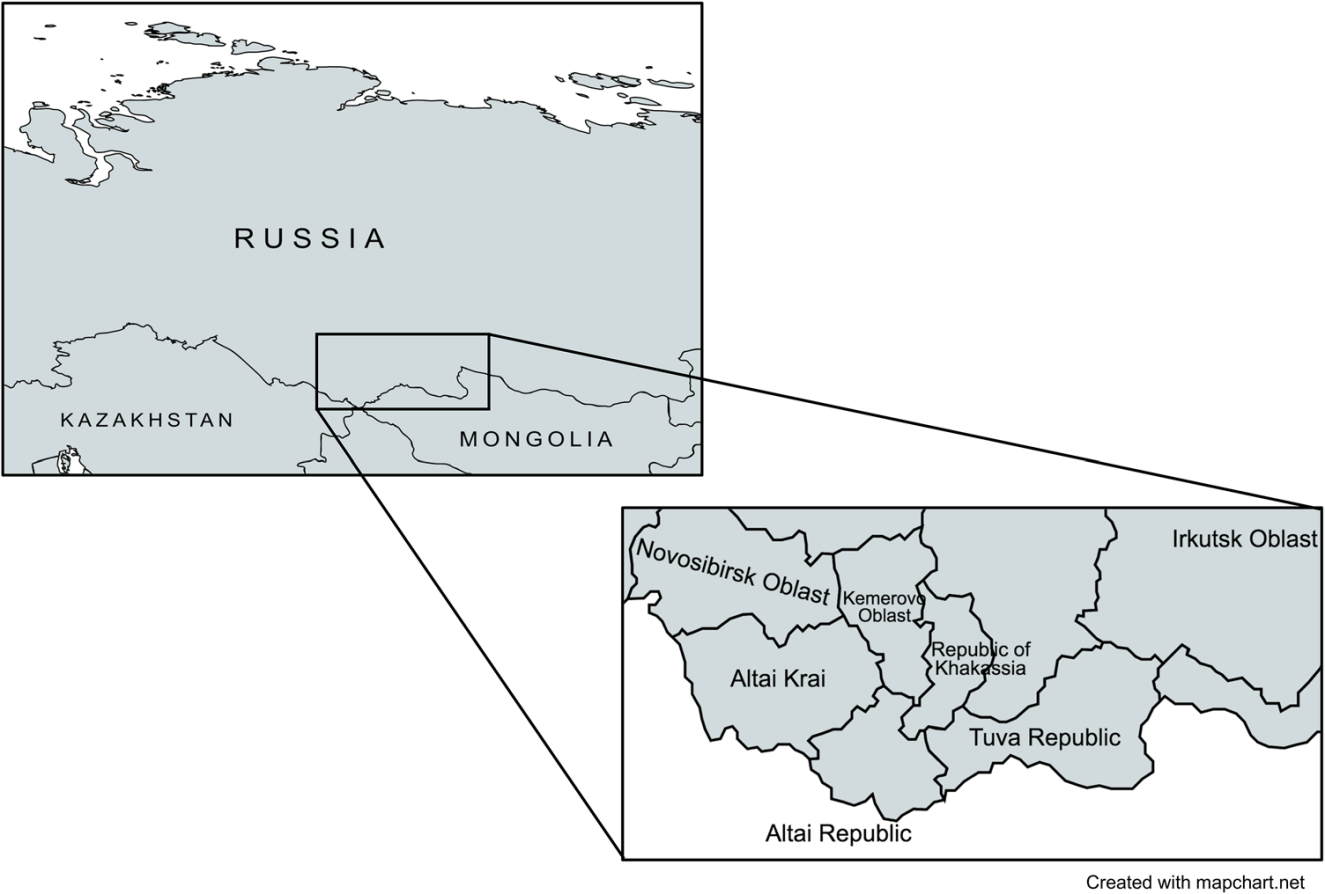
A map showing the location of Tuva, within Russia, where Mongolia borders Tuva’s south.

This study was performed in January 2020, within the Barun‐Khemchick District of the Republic of Tuva, south-central Siberia, Russia (Fig. 5). The study was approved by the Local-Ethical Committee (LEC) of the Research Institute of Medical and Social Problems and Management of the Republic of Tuva (NII MSPU RT) and was conducted in accordance with the Declaration of Helsinki.

### Participants

Twelve adults (9 males and 3 females; Table 1) completed the study. Participants were recruited via purposive sampling. Participants lived in yurts and practised nomadic pastoralism, although no migration took place during the observation period. The research was discussed with the volunteers in their native language (Tuvan), and written informed consent was obtained from the participants. The participants were from a total of 8 yurts. The participants included: three couples (male and female) from 3 yurts, two male relatives from 1 yurt, and males from 4 other yurts living alone or with their partners.

### Protocol

**Figure 6:**
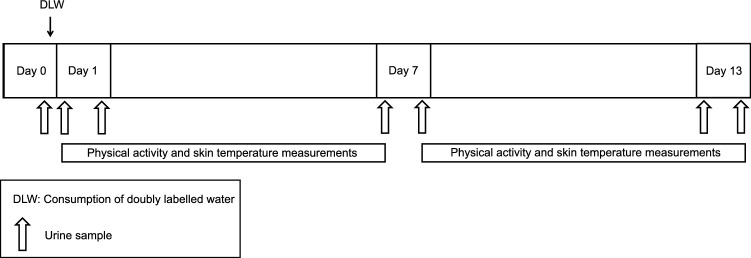
Protocol of the field work and measurements involved. DLW indicates consumption of the doubly labelled water. Urine sample collections are denoted by the larger arrows on Days 0, 1, 7, and 13.

Each participant was studied during a 14-day period (Fig. 6). The protocol consisted of two 6-day periods, following the Maastricht protocol for the doubly labelled water method^58^. Total energy expenditure (doubly labelled water), physical activity (accelerometery), skin temperatures (wireless temperature sensors), and the participant’s ambient temperature (wireless temperature sensor) were continuously measured. The wearable sensors were attached to participants on the morning of Day 1 (start of period 1) and collected on the morning of Day 7 (end of period 1) to download data. The sensors were reattached on the evening of Day 7 (start of period 2) for another 6 days of measurements and collected on the evening of Day 13 (end of period 2). In addition, lifestyle questionnaires were administered. Measurements were performed in the participant’s yurt to minimise disruption to their usual lifestyle. Resting energy expenditure was measured before the field observation period under laboratory conditions in the nearest town.

### Anthropometrics

Body mass (BM) was measured in underwear on an electronic scale (Seca Sensa 804) to the nearest 0.1 kg. Height was measured to the nearest 0.5 cm. Total body water (TBW; L) was determined with isotope dilution according to the Maastricht protocol^58^. Isotope abundances in urine were determined in duplicate with an isotope-ratio mass spectrometer (IRMS, Thermo Scientific Delta V Advantage, Thermo Fisher Scientific). Total body water (TBW) was calculated from the average of the oxygen-18 and deuterium dilution space^59^. Percentage fat-free mass (FFM) and fat mass (FM) were then calculated, assuming hydration of the FFM of 73%, as follows: FFM% = TBW/0.73 × 100%, and FM% = (BM-FFM)/BM × 100%.

### Energy expenditure

Resting energy expenditure was measured in the morning or in the afternoon after at least a 4-h fast to minimise diet-induced thermogenesis^37^. Participants refrained from consuming alcohol 24 h before the testing, and caffeine-containing drinks on the day of testing. REE was measured by indirect calorimetry using a face-mask system (Metalyzer 3B, Cortex). Prior to the measurements, calibration of the indirect calorimetry system was performed in the measurement room.

A cold room (9.4 ± 0.5 °C) was used for the REE measurement because additional tests were performed which are not reported in this paper. For the REE measurement, participants were in a supine position. A thermoneutral condition was created by having participants wear a thermal hat, thermal socks, underwear, shorts, singlet (females only). Also, the participants were covered with two duvets. Oxygen consumption and carbon dioxide production were measured continuously for 30 min. The initial 10 min and last 1 min of data were discarded, and the average of the remaining data were used to calculate REE. Energy expenditure was calculated according to Weir’s formula^60^.

Total energy expenditure was measured by the doubly labelled water technique according to the Maastricht protocol^58^. After the collection of a baseline urine sample on the evening of Day 0, subjects drank a weighed amount of a mixture of ^2^H_2_O and H_2_^18^O resulting in an initial excess body water enrichment of approximately 180 ppm for deuterium and 270 ppm for oxygen-18. Subsequent urine samples were collected from the second voiding in the morning on Day 1, and the evening on Day 1. Further urine samples were collected in the mornings and evenings of Days 7 and 13. The consumption of the doubly labelled water and the urine sample collections were supervised by a researcher at the participant’s yurt. The TEE of period 1 includes the measurement period of Days 1 to 7, whereas period 2 includes Days 7 to 13. PAL was calculated as TEE/REE.

Total energy expenditure was predicted for participants based on body mass (kg) and fat-free mass (kg). Sex-specific predictive equations for TEE based on body mass, in adults less than 65 years old, were created with data from developed countries that were rated as high on the human development index (HDI) (Appendix A^17^). The equations based on developed countries were: Males (42 cohorts, 1135 subjects) TEE = 0.0811 × BodyMass + 6.8827, and females (88 cohorts, 2492 subjects) TEE = 0.0421 × BodyMass + 7.2573. Additionally, sex-specific predictive equations for TEE based on body mass were created with data from developing countries that were rated as low or middle on the HDI (Appendix A^17^). The equations based on developing countries were: Males (9 cohorts, 136 subjects) TEE = 0.0867 × BodyMass + 6.3032, and females (13 cohorts, 339 subjects) TEE = 0.0499 × BodyMass + 6.4026. The predictive equation for TEE based on fat-free mass was TEE = EXP(0.708 × LN(FatFreeMass) − 0.391)^19^.

### Physical activity

Physical activity was determined using a triaxial accelerometer (wGT3X-BT, Actigraph). The activity monitor was attached to the participant’s waist in line with the right axilla, with an elastic belt. Participants were instructed to wear the activity monitor for the entire measurement period (2 times 6 days), including when sleeping, except for during water activities such as bathing. Adherence to wearing the activity monitor was checked verbally with the participants at the end of each 6-day period. The sample rate was 30 Hz. For analyses, data were converted into 60-s epochs. Initially, data were visually inspected for signs of non-wear, and if identified, the data were excluded from the analysis. The mean wear time for the activity monitor during period 1 and period 2 were 5.67 ± 1.16 d and 5.89 ± 0.25 d, respectively. In period 1, one monitor stopped recording prematurely, and during period 2 another monitor was not worn for one day. Vector magnitude (VM) was calculated according to: VM = √(total counts axis 1^2^ + total counts axis 2^2^ + total counts axis 3^2^). Troiano^40^ cut-off values were used to calculate time spent in different intensities of physical activity: sedentary (0–99 CPM), light activity (100–2019 CPM), moderate activity (2020–5998 CPM) or vigorous activity (> 5999 CPM). Mean minutes spent per day in each intensity category were then calculated.

### Skin temperature

Wireless temperature sensors (iButtons DS1992L, Maxim Integrated Products) were attached to the skin^61^ using semi-adhesive tape (Fixomull stretch) at the following four sites: left chest, left upper arm, right anterior thigh, and right lower leg^50^. The sensors were worn continuously, including when sleeping, except during water activities such as bathing. Additional adhesive tape and an explanatory figure showing the locations of the skin temperature sensors were provided to the subjects in case the sensors were removed. Adherence to wearing the temperature sensors was checked verbally with the participants at the end of each 6-day period. Additionally, data were visually inspected for signs of non-wear, and if identified, the data were excluded from the analysis. The sample rate was 5 min. Mean skin temperature was calculated according to Ramanathan^50^: *0.3* × *ChestT* + *0.3* × *UpperArmT* + *0.2* × *ThighT* + *0.2* × *LowerLegT*, where *ChestT* is the chest skin temperature (°C), *UpperArmT* is the upper arm skin temperature (°C), *ThighT* is the thigh skin temperature (°C), and *LowerLegT* is the lower leg skin temperature (°C).

### Environmental temperatures

A wireless temperature sensor (iButton DS1992L, Maxim Integrated Products) was attached to the outer surface of the clothing of the participants to determine the participant’s ambient temperature. Depending on the clothing, the sensor was attached with a clip or adhesive tape (Fixomull stretch). The sample interval was 5 min. Data were visually inspected for signs of non-wear and, if identified, the data were excluded from the analysis. For one subject, ambient temperature data had to be excluded due to non-wear. The thus obtained ambient temperature was measured during the day (07:00–19:00) of periods 1 and 2. Additionally, the duration of daytime ambient temperatures below 0 °C was calculated for both periods. The indoor and outdoor air temperatures of one yurt were assessed. The temperature inside the yurt was measured using a PT100 sensor (± 0.15 °C) connected to UX120-006 M (Onset HOBO) datalogger every 1 min, at the height of 0.9 m next to the bed of the participant. Outdoor air temperature (± 0.25 °C) was measured using a mobile weather station H21 (Onset HOBO), every 5 min, next to the entrance of the yurt, at the height of 2.7 m.

### Lifestyle questionnaire

Each participant was asked questions about their lifestyle in their native language. Questions were focused on the participant’s daily activities, sleep habits, the extent of participation in subsistence activities, and ownership of various consumer goods and livestock.

### Statistical analysis

Data were analysed using SPSS version 25 for Mac. Data are presented as mean ± SD unless stated otherwise. Differences between periods were assessed with a two-tailed paired t-test or Wilcoxon signed-rank test if data were non-normally distributed. The relationship between the participant’s mean skin temperature and daytime ambient temperature during the whole period was assessed with a linear mixed model. Restricted maximum likelihood was used as the estimation method. The dependent variable was mean skin temperature, the participants were assigned as a random effect with random intercept, and the ambient temperature, sex, and their interaction were included as fixed effects. A chi-square difference test was used to determine if including participants as a random effect significantly improved the model. The relationships between body fat % and mean skin temperature during period 1 and period 2 were assessed with the Pearson correlation. Measured TEE and TEE predicted by body mass, and fat-free mass were compared with one-way repeated measures ANOVAs. If the assumption of sphericity was violated, then the Greenhouse–Geisser method was used. When a significant main effect was found, paired t-tests were used to locate any differences. The relationships between variables were assessed with simple or multiple linear regression. Statistical significance was accepted when P < 0.05.

## Data Availability

Data are available upon reasonable request.
